# Hierarchy of the Components in Spray-Dried, Protein-Excipient Particles Using DNP-Enhanced NMR Spectroscopy

**DOI:** 10.1021/acs.molpharmaceut.3c00539

**Published:** 2023-10-02

**Authors:** Pierrick Berruyer, Maria Lindkvist, Sandra Gracin, Tatiana Starciuc, Andrea Bertarello, Baptiste Busi, Staffan Schantz, Lyndon Emsley

**Affiliations:** † Institut des Sciences et Ingénierie Chimiques, Ecole Polytechnique Fédérale de Lausanne (EPFL), CH-1015 Lausanne, Switzerland; ‡ Inhalation Product Development, Pharmaceutical Technology & Development, Operations, AstraZeneca, SE-431 83 Mölndal, Sweden; § Oral Product Development, Pharmaceutical Technology & Development, Operations, AstraZeneca, SE-431 83 Mölndal, Sweden

**Keywords:** NMR spectroscopy, dynamic nuclear polarization, protein drug formulations, structure

## Abstract

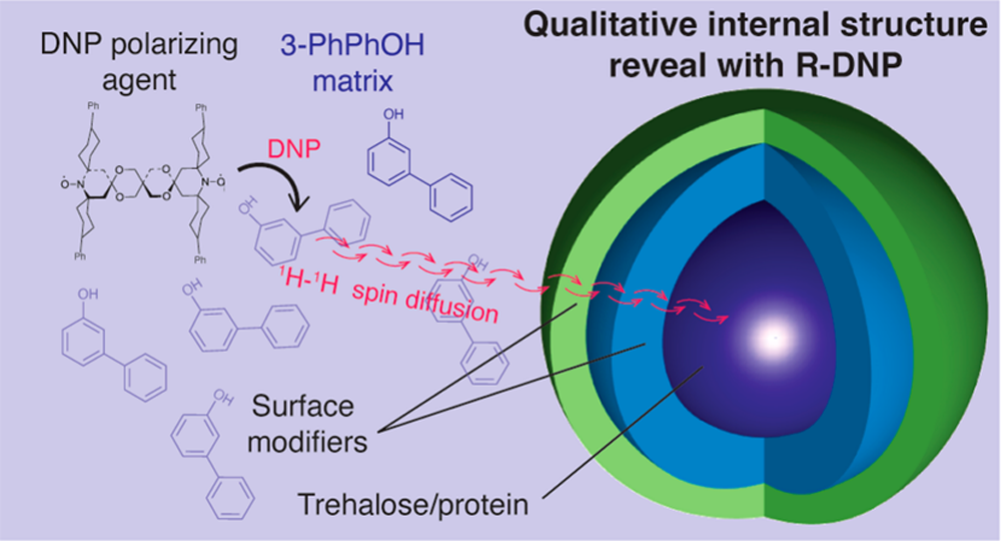

Protein-based drugs are becoming increasingly important, but there are challenges associated with their formulation (for example, formulating stable inhalable aerosols while maintaining the proper long-term stability of the protein). Determining the morphology of multicomponent, protein-based drug formulations is particularly challenging. Here, we use dynamic nuclear polarization (DNP) solid-state NMR spectroscopy to determine the hierarchy of components within spray-dried particles containing protein, trehalose, leucine, and trileucine. DNP NMR was applied to these formulations to assess the localization of the components within the particles. We found a consistent scheme, where trehalose and the protein are co-located within the same phase in the core of the particles and leucine and trileucine are distributed in separate phases at the surface of the particles. The description of the hierarchy of the organic components determined by DNP NMR enables the rationalization of the performance of the formulation.

## Introduction

Inhaled drugs are becoming increasingly important. For example, in the emerging field of inhaled biologics, proteins and peptides have attracted considerable attention in current biotechnology development.^1^ Current efforts toward protein formulation manufacturing focus on securing an inhalable aerosol in combination with formulations and manufacturing processes that ensure the proper long-term stability of the protein. The dispersion of proteins in sugar/polyols matrices, such as trehalose or mannitol, is a well-established technique for stabilizing proteins in dry and room temperature environments while preserving their structural integrities and functions.^2,3^ A relatively new emerging approach in drug delivery, for the use of therapeutic proteins in particular, is based on spray-drying.^4−7^ In essence, the ingredients are dissolved in a solvent to afford a concentrated solution, and the latter is then sprayed into very small droplets typically using an atomizer; finally, the droplets are quickly dried using a hot gas flow. Amino acids, including leucine, have been reported to tune the dispersibility and the aerosolization of the final product.^8,9^ Lechuga-Ballesteros et al. showed that trileucine improves the aerosol performance and the physical stability of the spray-dried materials.^10^ Such multicomponent spray-dried protein formulations raise fundamental structural questions about the final materials. In particular, the morphology of the solid particles, i.e., the spatial distribution of the different components in the final material and the homogeneity of the different phases, is still subject to discussion. So far, the characterization of such particles typically relies on scanning electron microscopy (SEM), laser light scattering to obtain the particle size distribution (PSD), and the external aspect of the particles.^10^ The combination of these techniques with X-ray photoelectron spectroscopy (XPS) helps to understand the chemical composition at the surface or near the surface of the particles.^10^ The internal composition is mostly inferred from the measured solubilities of the components in the solution used for spray-drying. It is usually assumed that components with lower solubility will precipitate earlier during the drying process.^10^ Here, we show how solid-state NMR spctroscopy in combination with the hyperpolarization method dynamic nuclear polarization (DNP) has the potential to determine the distribution of the components in the final spray-dried particles. Thereby we provide a more reliable image of the internal composition of the particles.

Briefly, DNP is a hyperpolarization approach in NMR consisting of transferring electron spin polarization to nuclear spins, which can lead to an increase in NMR sensitivity by up to 2 orders of magnitude.^11,12^ To perform DNP of materials, samples are typically impregnated with a solution containing a DNP polarizing agent, forming a polarizing phase localized at the surface of the material.^13^ Hyperpolarization is first generated within the solvent phase upon microwave irradiation and is then transported through the material by spontaneous ^1^H–^1^H spin diffusion (SD) to achieve hyperpolarization of the bulk material.^14^ The potential of DNP to study pharmaceutical formulations is clear.^15−17^ Particularly, in the context of formulations, the transport process of the polarization from the surface solvent layer to the core of the material allows for the probing of a material from its surface to its core on a nano- to micrometer length scale. This experiment, denoted relayed DNP (R-DNP),^14,18,19^ has been used to measure domain sizes in microcrystalline particles, porous materials, and multicomponent cellulosic samples.^14^ R-DNP has also been used to describe the core–shell structure of organic crystalline nanoparticles^20^ and to describe the structure of lipid nanoparticles (LNPs) containing siRNA or mRNA, where it was used to localize the different components of the LNPs.^17^ Very recently, Berruyer et al. showed that R-DNP can be used to perform radial imaging of complex particles.^21^

In the present work, we used dynamic nuclear polarization solid-state NMR spectroscopy to describe the hierarchy of components within spray-dried particles of protein formulations. Namely, we prepared different spray-dried formulations of an AstraZeneca protein development compound with trehalose, leucine, and trileucine. DNP NMR was then applied to these formulations to assess the spatial distribution of the components within the particles. Different sets of particles were synthesized and analyzed while varying their compositions. We found a consistent scheme where trehalose and the protein are co-located within the same phase in the core of the particles and leucine and trileucine are distributed in separate phases at the surface of the particles.

## Materials and Methods

### Materials

The composition of the different formulations prepared is reported in Table 1. They are divided into three categories. In category (i), the spray-dried particles are placebos containing varying amounts of trehalose and leucine. In category (ii), the spray-dried samples are prepared with the protein, trehalose, and leucine. Note that the presence of the buffer is concomitant with the associated PBS buffer in the protein solution. Samples ii.1, ii.2, and ii.3 have similar compositions but different manufacturing conditions, leading to different particle morphology with the same composition. The category (iii) samples are prepared with the protein, trehalose, leucine, and trileucine.

**Table 1:** Composition of the Different Samples Reported in Total Weight Percentage (%)

	ref	AZ protein	buffer	trehalose	leucine	trileucine	comment
i	i.1	-	-	55	45	-	-
	i.2	-	-	75	25	-	-
	i.3	-	-	85	15	-	-
ii	ii.1	60	10.6	9.4	20	-	D50 = 1.92 μm
	ii.2	60	10.6	9.4	20	-	D50 = 2.3 μm
	ii.3	60	10.6	9.4	20	-	D50 = 6.22 μm
	ii.4	2	0.4	77.6	20	-	-
	ii.5	60	10.6	29.4	-	-	-
iii	iii.1	10	1.77	75.63	10	2.6	local labeling ^13^C, ^15^N
	iii.2	10	1.77	65.63	20	2.6	local labeling ^13^C, ^15^N
	iii.3	10	1.77	76.73	10	1.5	local labeling ^13^C, ^15^N

The excipients trehalose, l-leucine, and trileucine were all of pharmaceutical grade (PhEur or NF and USP). The ^15^N-labeled l-leucine (98 atom % ^15^N) was purchased from Aldrich. Fully ^13^C-labeled trileucine (>95% HPLC purity) was custom-made by Bachem AG (Switzerland).

For each sample, the following procedure was followed: an aqueous solution containing the desired weight amount in the excipient and active molecules of the dry particle described in Table 1 was prepared. The latter was then used in a Büchi 290 spray-drier to produce a fine mist and dry it. The spray-drying parameters were adjusted using standard procedures^22−26^ in order to afford the final formulations and particle sizes given in Table 1.^27,28^

### DNP NMR Spectroscopy

DNP-enhanced solid-state NMR experiments were performed using a 9.4 T Bruker Avance III HD NMR spectrometer equipped with a 3.2 mm LTMAS DNP probe in triple resonance mode ^1^H/^13^C/^15^N. The probe operated at sample temperatures of about 100 K using a Bruker LTMAS cabinet. Continuous microwave irradiation was generated using either a 264 GHz Bruker klystron or a 263 GHz Bruker gyrotron. In each case, the main magnetic field of the NMR spectrometer was finely adjusted to match the cross-effect maximum intensity of the DNP agent TEKPOL.^29^ The sample was spun at the magic angle at 12.5 kHz during signal averaging (except when otherwise indicated). Detailed NMR experimental parameters are provided in Tables S1 and S2.

## Results and Discussion

### Principles of Component Distributions in Particles from R-DNP

**Figure 1:**
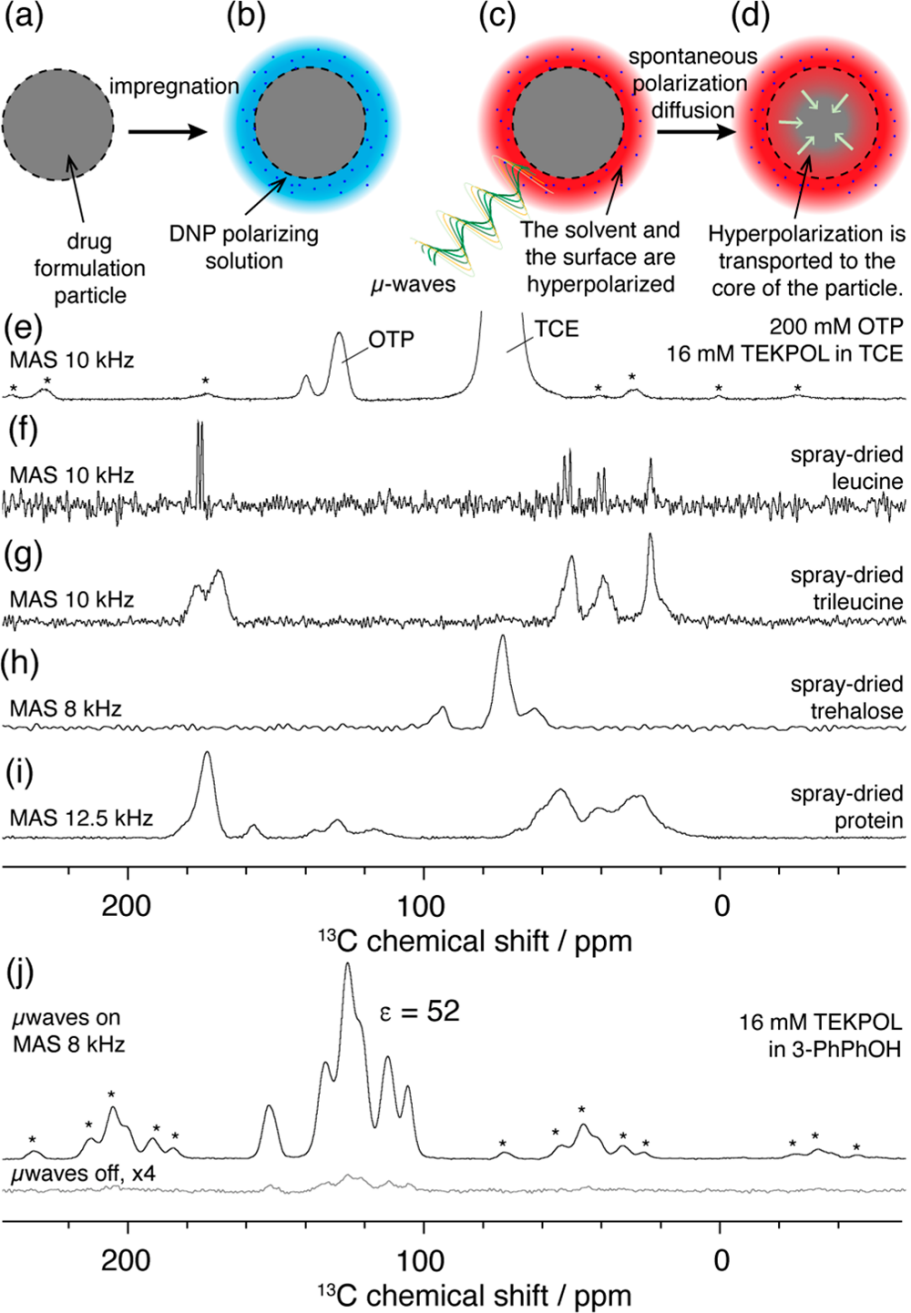
(a–d) Illustration of the R-DNP method: the dry powder from the AZ formulations (a) is first impregnated with a solution containing the DNP polarizing agent, affording (b) a layer of polarizing solution at the surface of each grain from the powder sample. (c) Once inserted in the cold (100 K) DNP probe, the frozen polarizing layer is hyperpolarized upon application of continuous μ-waves. (d) Finally, the hyperpolarization is spontaneously transferred from the surface to the core of the particle, mediated via ^1^H spin diffusion. (e–i) ^1^H–^13^C CPMAS NMR spectra of the different formulation components. Spectra have been recorded at 100 K with a magnetic field of 9.4 T on spinning samples, and the MAS rate is reported for each spectrum. Sample (e) is recorded under μ-wave irradiation to yield a DNP sensitivity enhancement. Samples (f–i) are dry (non-impregnated) materials, recorded without μ-wave irradiation. (j) ^1^H–^13^C DNP CPMAS NMR of 16 mM TEKPOL in flash-frozen 3-PhPhOH, recorded at ca. 100 K with and without μ-waves.

Figure 1 schematizes the dynamics of hyperpolarization in a R-DNP experiment. For the study of the formulations here, R-DNP is performed using a polarizing solution containing the stable organic radical TEKPOL.^29^ As shown in Figures 1a and b, the material is impregnated with the radical solution. The experiments are performed in a spinning sapphire rotor in a NMR probe working at temperatures of approximately 100 K. At this temperature, the solvent forms a glass where the polarizing agent is uniformly distributed. Then, as depicted in Figure 1c, the sample is continuously irradiated with microwaves (μ-waves) which allows the (partial) transfer of the unpaired electron spin polarization to the ^1^H nuclei of the frozen solvent. In our conditions, the solvent is almost instantaneously polarized.^30^ As illustrated in Figure 1d, the ^1^H hyperpolarization generated in the solvent spontaneously diffuses into the organic material through a process called spin diffusion. The slow diffusion of the hyperpolarization within the bulk particle gives the opportunity to probe the internal structures of the particles. As demonstrated in ref (31), from numerical spin diffusion simulations it is possible to qualitatively determine if the different components of the particles share the same phases or occupy different phases in the particles and to determine which component is closer to the surface or the core of the particle if they are in different phases. Under the conditions of the experiments, ^1^H spectra do not allow for the direct measurement of the steady-state DNP enhancement of each component because of resolution. Thus, ^1^H polarization is usually transferred via cross-polarization (CP) to a heteronucleus (e.g., ^13^C, ^15^N···). Finally, the DNP enhancement is obtained by taking the ratio of the component signal area measured with and without μ-wave irradiation.

### Sample Impregnation for R-DNP

The R-DNP experiments require impregnation of the materials with a solvent containing the DNP polarizing agent. This impregnation technique must respect the following prerequisites: the solvent does not dissolve the observed materials, the solvent must not change the nature of any component of the materials, and the solvent should wet the surface well enough to allow an efficient transfer of polarization from the frozen solvent phase to the material.

As shown in Table 1, the samples were grouped into three categories. Materials from categories (i) and (ii) were impregnated using the typical 16 mM TEKPOL in 1,1,2,2-tetrachloroethane (TCE) solution.^32^ An additional 200 mM orthoterphenyl (OTP) was dissolved in the TCE to allow unambiguous measurement of the DNP enhancement of the solvent phase. The use of TCE was found compatible with trehalose, leucine, and the drug; TCE did not dissolve any of the three components (to evaluate solvent compatibility, each component was impregnated with the candidate solvent. If the solid dissolved, then the solvent was invalidated. If the solid did not dissolve, a ^13^C solid-state NMR spectrum was recorded and compared with the spectrum of the neat solid to ensure no phase change had been induced).

Materials from category (iii) were impregnated with 16 mM TEKPOL in 3-phenylphenol. As shown in Figures S1a and b, we found that TCE induced a phase transition of a pure spray-dried trileucine sample, clearly converting from an amorphous structure to a more organized structure upon impregnation with TCE. To prevent the phase transition, other DNP solvents were tested. Dibromoethane^33^ and toluene–CD_3_ were found to dissolve trileucine. We tested a more advanced DNP sample preparation using an OTP solvent.^34,35^ OTP is a crystalline solid at room temperature. Thus, the TEKPOL polarizing agent in an OTP solution is typically prepared by dissolving the two compounds in chloroform, and the chloroform is then evaporated. The obtained powder is then mixed with the solid particles and packed in the rotor, which is then heated at ca. 70 °C to melt the OTP and eventually wet the solid sample particles.^34,35^ The rotor is then quickly frozen at 100 K upon insertion into the precooled DNP probe. The flash-freezing process allows for the formation of an amorphous TEKPOL/OTP polarizing phase on the target particles. While, as reported in Figure S1c, the trileucine structure is preserved in such a formulation with OTP, we found very poor polarization transfer from the polarizing TEKPOL/OTP phase to the materials; while a DNP enhancement of 145 was achieved in the polarizing phase, the trileucine enhancement was found to be only 7.5. We attributed this observation to the probable poor wetting of the particle by the OTP, preventing efficient polarization transfer.

Thus, we introduce here the use of 3-phenylphenol (3-PhPhOH) as a DNP solvent. Comparable to OTP,^34,35^ 3-PhPhOH is a solid at room temperature; an, the sample can be prepared in a similar way as for OTP impregnation: TEKPOL and 3-phenylphenol are dissolved in chloroform, and the latter is then evaporated. The obtained powder is then mixed with the solid particles and packed in the rotor, which is then heated to 90 °C to melt the 3-PhPhOH and wet the solid sample particles, and the rotor is flash-frozen. As shown in Figure 1j, we found that 16 mM TEKPOL in 3-PhPhOH without any material (bulk solution) can give a solvent enhancement of a factor of 52. The trileucine does not suffer from a phase transition from impregnation with 16 mM TEKPOL in 3-PhPhOH (see Figure S1d). Moreover, while impregnation with 16 mM TEKPOL in OTP 95%-d_8_ was not concomitant with high hyperpolarization of the impregnated trileucine, using 16 mM TEKPOL in 3-PhPhOH allowed good transfer of polarization from the solvent phase to the materials. We attributed that to the enhanced hydrophilicity of 3-PhPhOH compared to OTP, increasing surface wetting (here we note that impregnation with 3-PhPhOH at 90 °C might induce a change in the protein structure, but we assume that heating the particles for short times does not induce a complete reorganization of the particles; i.e., each component (including the protein) will remain in the same location within the particle, not changing the internal hierarchy).

All in all, these impregnation strategies allowed to perform R-DNP studies in the three identified categories of materials.

### Determining the DNP Enhancements of the Different Components

Another prerequisite to implement a R-DNP strategy is the ability to independently measure the DNP enhancements of the different components of the material, in this case, the active ingredient protein, trehalose, leucine, and trileucine. Figures 1f–i represent the ^1^H–^13^C CPMAS NMR spectra of the four different pure and spray-dried compounds. The aspect of the ^13^C signals suggested that for the pure spray-dried compounds, leucine is crystalline, whereas trileucine and trehalose are amorphous. Note that this might not be the case for the mixtures.

Samples of category (i) were impregnated with 16 mM TEKPOL and 200 mM OTP in TCE. The leucine signal at 180 ppm and trehalose at 95 ppm can be integrated independently and provide the DNP enhancement of the two components. As illustrated in Figures 1e and h, the highest intensity trehalose signal will overlap with the solvent TCE peak at 74 ppm. Because of trehalose/TCE overlap, 200 mM OTP was dissolved in the TCE and the OTP signal at 125–145 ppm was used to access the solvent DNP enhancement.

Samples of category (ii) were impregnated with 16 mM TEKPOL and 200 mM OTP in TCE. ^13^C signals at 160 ppm of the protein and at 95 ppm of the trehalose allow the obtention of the DNP enhancement of the two components (Figures 1h and j). The solvent enhancement is obtained from the dissolved OTP in the TCE (Figure 1e). Finally, we can note that all leucine signals overlap with the drug peaks (Figures 1f and j). To extract the leucine DNP enhancement, a difference spectroscopy strategy was implemented. The enhanced spectra of both the formulation and the pure protein were recorded in similar conditions. Then, the spectrum of the drug was scaled to match the intensity of the resolved drug peak at 160 ppm of the formulation. Finally, the difference between the two spectra was computed and allowed for the integration of the leucine peak at 20 ppm.

Adding trileucine to the mixture gave an extra level of complexity. Indeed, trileucine is a trimer of leucine; thus, the chemical shifts of the two compounds are similar, and there is no spectral resolution between the two chemicals (Figures 1f and g). Thus, for formulations mixing leucine and trileucine, a specific labeling strategy was used: the carbonyls of trileucine ^13^C=O were ^13^C-enriched, and leucine was ^15^N-enriched. Thus, on one hand, ^1^H–^15^N CPMAS NMR allows for the measurement of the leucine DNP enhancement. On the other hand, the ^13^C peak at 180 ppm from ^1^H–^13^C CPMAS will result not only from the trileucine-labeled carbonyls ^13^C=O but also from leucine and the drug. Considering, for example, sample iii.1, the ratio of ^13^C signal intensities would be
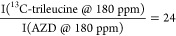

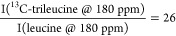
Thus, we can make the assumption that ^13^C-trileucine dominates the 180 ppm peak and allows for the direct evaluation of the trileucine DNP enhancement. Trehalose DNP enhancement is measured using the resolved ^13^C signal at 75 ppm. Finally, the protein DNP enhancement cannot be measured independently due to either signal overlap with another component or the broad ^13^C 3-phenylphenol signal.

### Spatial Hierarchy of the Components from Steady-State DNP Enhancements

In order to describe the hierarchy of the different components of the particles, we measured the steady-state DNP enhancement of the different components in a R-DNP experiment. Components in the same phase will share the same steady-state DNP enhancement, whereas they would be different if the two components were in separate phases. Moreover, the physics of the spin diffusion shows that the steady-state enhancement reveals the hierarchy of the component in the particles.^17,31^ Typically, a core–shell particle with component 1 in the shell and component 2 in the core will give
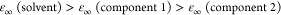
Theoretical development of R-DNP models, on which this study capitalized, has been detailed previously.^14,16,17,20,30,36^ In particular, Berruyer et al. introduced and demonstrated the classification of the components within sample particles by R-DNP methods.^31^

**Figure 2:**
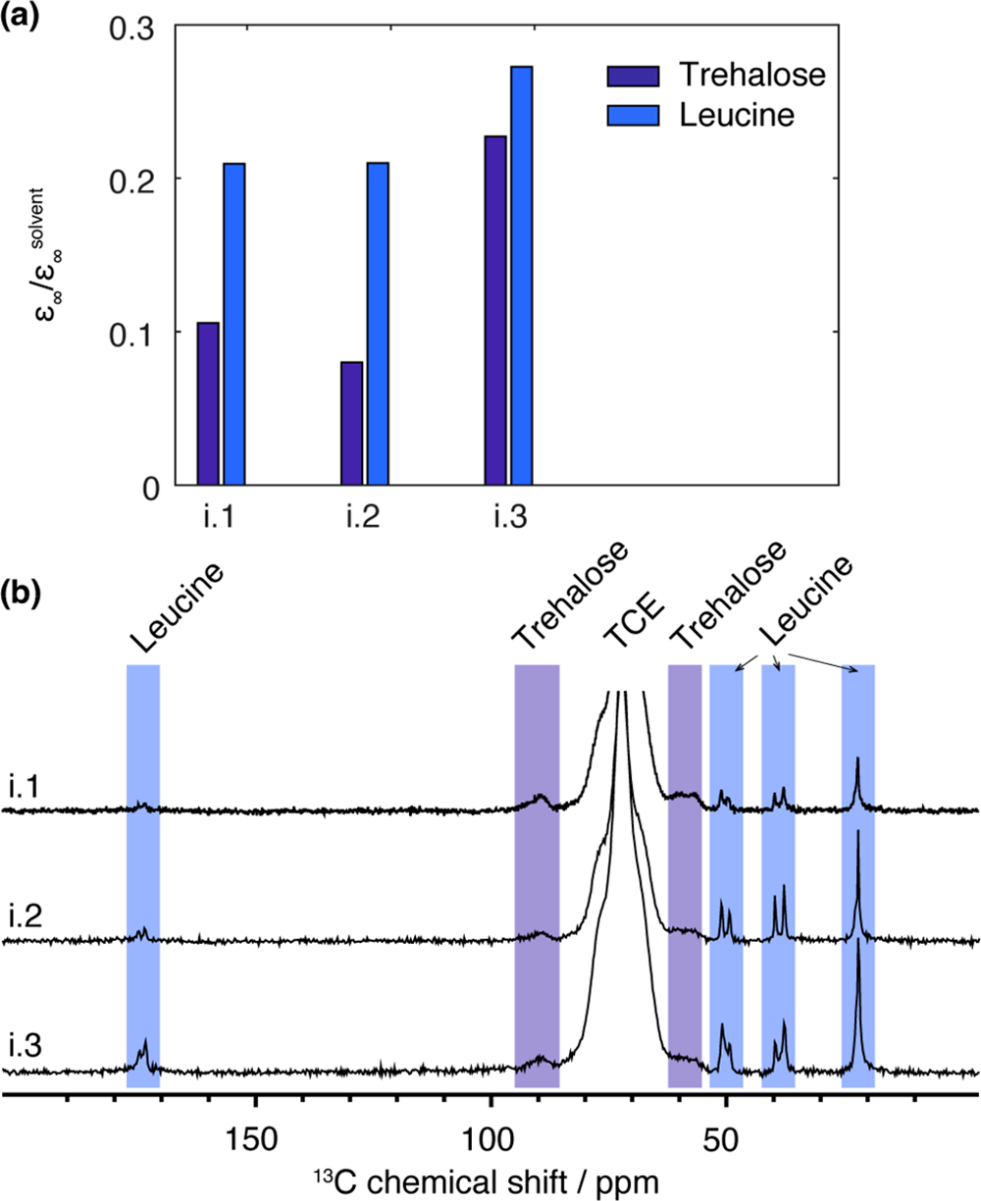
(a) Steady-state DNP enhancement (reported relative to the solvent enhancement) of the category (i) samples. (b) ^1^H–^13^C DNP CPMAS of the category (i) samples impregnated with 16 mM TEKPOL in TCE, recorded under μ-waves. The integrated regions to measure the different ^1^H enhancements are indicated by colored bands. From the signal-to-noise ratio of the NMR spectra, we estimated the average error on the DNP enhancement to be ±1. Error bars are not shown as they would be too small to see on the scale of the figure.

Figure 2a reports the steady-state DNP enhancements measured on the three samples of category (i). The DNP enhancements are normalized to the solvent enhancement. We found that, regardless of the trehalose:leucine ratio within the samples, the DNP enhancement followed the order:
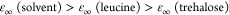
Thus, we find that in these spray-dried particles, leucine and trehalose are located in different phases, with particles being composed of a core of trehalose covered by a leucine layer. Figure 2b shows the ^1^H–^13^C DNP CPMAS NMR spectra of the three studied placebo formulations. The broad triplet peak between 65 and 80 ppm is assigned to the TCE solvent and overlapping with the trehalose peak. The peak at 90 ppm is assigned to trehalose. All the other peaks can be assigned to leucine. The ^13^C peaks of leucine at 39 and 50 ppm have a line width of ∼50 Hz, which suggests that the leucine phase is partly crystalline. A closer analysis of the leucine signal at 22 ppm shows the overlap of a dominant narrow peak with a line width of ∼40 Hz and a broader peak with a line width of ∼150 Hz, which accounts for the three final carbons of the leucine side chain (two CH_3_ and one CH).^37^

**Figure 3:**
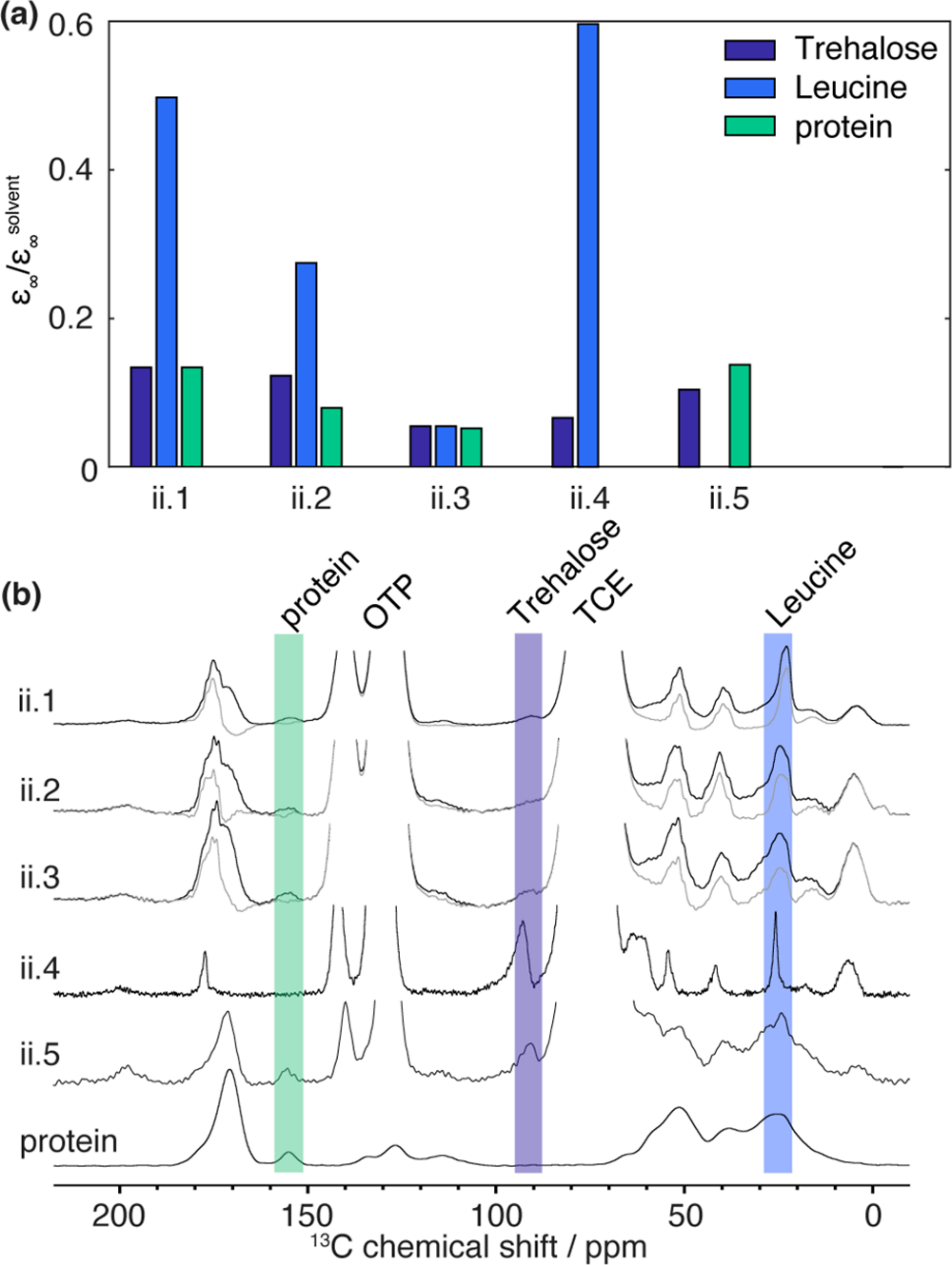
(a) Steady-state DNP enhancement (reported relative to the solvent enhancement) of the category (ii) samples. (b) ^1^H–^13^C DNP CPMAS of the category (ii) samples impregnated with 16 mM TEKPOL and 200 mM OTP in TCE recorded under μ-wave irradiation and of the pure drug at low temperature. For samples ii.1, ii.2, and ii.3, the gray lines indicate the ^13^C spectra obtained after subtraction of the pure drug spectrum (see above) to access the ^1^H DNP enhancement of leucine. The integrated regions to measure the different ^1^H enhancements are indicated by colored bands. From the signal-to-noise ratio of the NMR spectra, we estimated the average error on the DNP enhancement to be ±1. Error bars are not shown, as they would be too small to see on the scale of the figure.

Figure 3a presents the steady-state DNP enhancement measured on the class (ii) materials containing drug/trehalose/leucine components. Samples ii.1, ii.2, and ii.3 have the same composition, i.e., 60% protein, 10.6% PBS buffer, 9.4% trehalose, and 20% leucine. To prepare those materials, different spray-drying conditions were used, thus leading to different particle diameters reported in Table 1. Due to the increase in particle size from ii.1 to ii.3, one can expect lower DNP enhancement for ii.3, and this is what is observed: the general level of hyperpolarization *ε*_∞_/*ε*_∞_^solvent^ for the three ingredients decreases. This is due to the longer distance of hyperpolarization travel required to polarize larger particles.^14^ For the three samples, we observe *ε*_∞_ (protein) = *ε*_∞_ (trehalose), showing that the protein forms a single phase with the stabilizer trehalose in the spray-dried formulations. For the smaller particles ii.1 and ii.2, the following order is observed:
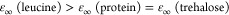
As a consequence, we can conclude that, similarly to what we observed from class (i) (placebo particles), leucine is located in a phase at the surface of the protein/trehalose cores. Note that the results show the existence of this phase, but we cannot conclude whether it is a pure leucine phase or a phase containing a higher concentration of leucine than the core. For the bigger particles ii.3, we obtained:
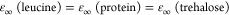
It shows that in this case leucine mixes with the stabilized drug/trehalose phase.

The formulations ii.4 and ii.5 have been prepared with similar spray-drying conditions but different compositions, as detailed in Table 1. Unfortunately, for sample ii.4, the very small quantity of protein (2 wt %) in the sample made it not possible to evaluate the *ε*_∞_ (protein). Also, note that ii.5 does not contain any leucine, explaining why Figure 3a does not report a value.

For sample ii.5, similar to the previously discussed sample, we found that *ε*_∞_ (protein) = *ε*_∞_ (trehalose), showing that the protein forms a single phase together with the stabilizer trehalose in the spray-dried formulations. Moreover, for sample ii.4, we observed *ε*_∞_ (leucine) > *ε*_∞_ (trehalose), showing that the leucine forms a layer on the trehalose core of the particle. Note that in the case of sample ii.4, the protein was not detected (due to very low concentration of the protein in that sample). All in all, the conclusions drawn for these two samples are consistent with the first three formulations of class ii.

Figure 3b reports the DNP-enhanced ^1^H–^13^C CPMAS NMR spectra of the class (ii) samples (Figure 3b, black lines). For samples ii.1–ii.3, as described above, the determination of the leucine DNP enhancement is enabled by subtracting the ^1^H–^13^C CPMAS NMR spectrum of the pure protein from the spectrum obtained with the formulation. The resulting difference spectra are plotted for samples ii.1–ii.3 in Figure 3b with gray lines. The leucine peak at 22 ppm is used for the determination of *ε*_∞_ (leucine), reported in Figure 3a. In the three formulations, samples ii.1–ii.3, the leucine peaks likely indicate more disordered leucine phases in contrast to samples of class (i), where the leucine peaks were indicative of a crystalline nature for the leucine phase. Nonetheless, it should be noted that the difference spectroscopy strategy used here potentially introduced significant peak distortions, and thus the sole aspect of the leucine peak might not be sufficient to unambiguously make conclusions about the nature of the leucine phase. In the case of sample ii.4, the width of the leucine peak at 22 ppm is ∼140 Hz, indicating a potentially more ordered nature of the component in the particle shell as it is close to the width measured on crystalline leucine samples.

**Figure 4:**
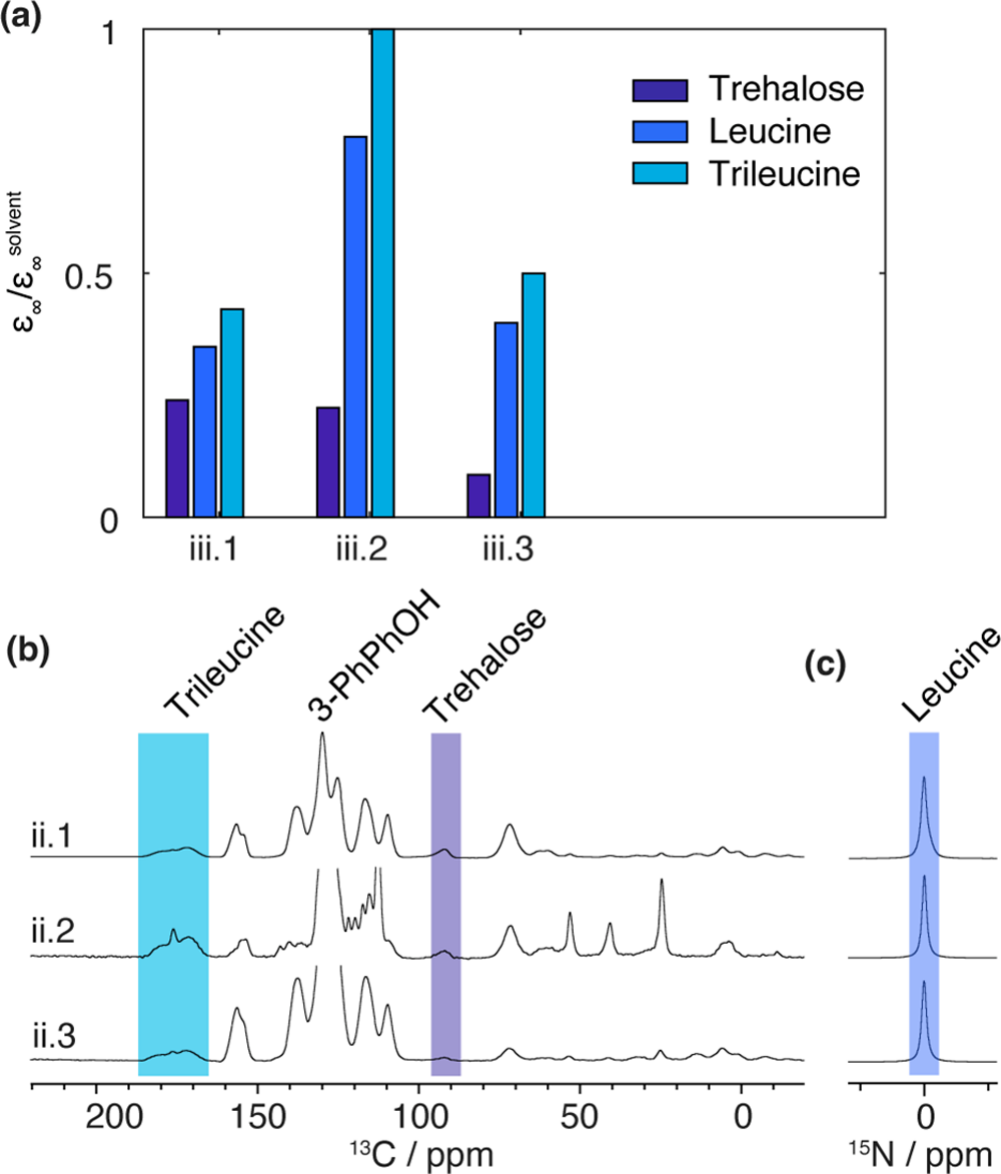
(a) Steady-state DNP enhancement (reported relative to the solvent enhancement) of category (iii) samples. (b) ^1^H–^13^C and (c) ^1^H–^15^N DNP CPMAS spectra of the category (iii) samples impregnated with 16 mM TEKPOL in 3-PhPhOH recorded under μ-wave irradiation. The integrated regions to measure the different ^1^H enhancements are indicated by colored bands. The specific ^13^C enrichment of trileucine and ^15^N enrichment of leucine (see the text) allow the differentiation of the two components. From the signal-to-noise ratio of the NMR spectra, we estimated the average error of the DNP enhancement to be ±1. Error bars are not shown as they would be too small to see on the scale of the figure.

Figure 4 presents the steady-state DNP enhancement measured on the class (iii) materials containing drug, trehalose, leucine, and trileucine components. As explained above, to allow spectral distinction between leucine and trileucine, a specific labeling strategy was implemented. The leucine is ^15^N-labeled. The trileucine is ^13^C-labeled on ^13^C=O sites. Although this strategy allowed for the independent measurement of *ε*_∞_ (leucine) and *ε*_∞_ (trileucine), it does not allow the obtention of *ε*_∞_ (protein). In the previous section, regardless of the sample, we systematically observed that *ε*_∞_ (protein) = *ε*_∞_ (trehalose), showing that the drug and the trehalose are located in a common phase. Then, here, we are making the assumption that this result can be generalized to all the samples of the present section.

For the three samples, the following order is always observed:
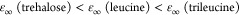
Thus, it establishes the following hierarchy: the core is composed of a mixture of trehalose and protein, and the latter is then covered with a layer of leucine, which is then covered with a shell of trileucine.

## Conclusion

To conclude, the hierarchy of the formulation components in spray-dried powder particles containing an AZ protein development compound, trehalose, leucine, and trileucine was determined using solid-state NMR enhanced by dynamic nuclear polarization. In all of the formulated samples, we found that the drug forms a single phase together with trehalose in the core of the particles. The amino acids then form layers on top of the drug/trehalose core. The determined structure is in agreement with claims that amino acids increase the dispersibility and the aerosolization of spray-dried particles.^8−10^ These results are more generally in line with expectations, as the amino acids are expected to form an outer layer either based on Peclet numbers^38^ or, as discussed by Ordoubadi et al.,^39^ resulting from higher surface activity and lower solubility. In the samples containing leucine and trileucine, we first found a leucine layer closer to the core and then a trileucine layer at the surface of the particles. Our results confirm the relevance of DNP NMR in this context, providing a powerful method to study pharmaceutical formulations at the micro- and mesoscopic scale, both of which are extremely relevant to rationalize properties of formulations. The DNP NMR method developed here is particularly relevant in the context of all-organic particles, where the hierarchy of internal composition is hard to access with other techniques. As discussed in the main text, the major condition required to perform these DNP NMR measurements is to find a compatible, non-solvent polarizing solution to generate hyperpolarization and transfer it to the pristine particles. Here, this was done by the introduction of a new DNP solvent, 3-phenylphenol.

## Supporting Information Available

Experimental details, supplementary figure (PDF)
