# Supplementary material for: Hierarchy of the Components in Spray-Dried, Protein-Excipient Particles Using DNP-Enhanced NMR Spectroscopy
Source: Mol Pharm. 2023 Oct 2;20(11):5682–9. doi: 10.1021/acs.molpharmaceut.3c00539 (PMC10630941; doi:10.1021/acs.molpharmaceut.3c00539)
Supplement: Supplementary file 1 — mp3c00539_si_001.pdf [file mp3c00539_si_001.pdf]

Supporting Information

Hierarchy of the components in spray-dried protein-excipient particles using DNP Enhanced NMR Spectroscopy.

Pierrick Berruyer,[a] Maria Lindkvist,[b] Sandra Gracin,[b] Tatiana Starciuc, [b] Andrea Bertarello,[a] Baptiste Busi,[a] Staffan Schantz[c]* and Lyndon Emsley[a]*

[a] Institut des Sciences et Ingénierie Chimiques, Ecole Polytechnique Fédérale de Lausanne (EPFL), CH-1015 Lausanne, Switzerland

[b] Inhalation Product Development, Pharmaceutical Technology & Development, Operations, AstraZeneca, SE-431 83 Mölndal, Sweden

[c] Oral Product Development, Pharmaceutical Technology & Development, Operations, AstraZeneca, SE-431 83 Mölndal, Sweden

*corresponding author: staffan.schantz@astrazeneca.com, lyndon.emsley@epfl.ch

Supplementary figure

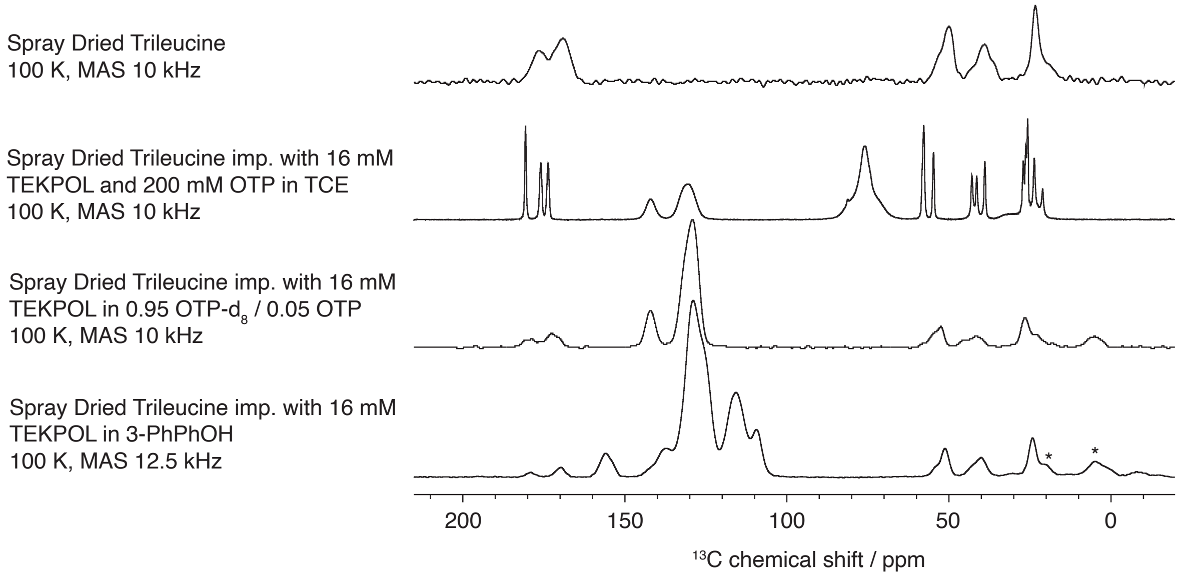

Figure S1. ^{1}H-^{13}C CPMAS NMR spectrum of spray-dried trileucine in different conditions: (a) dry pristine spray-dried trileucine powder. The spray-dried trileucine powder was impregnated with (b) 16 mM TEKPOL and 200 mM OTP in TCE; (c) 16 mM TEKPOL in OTP-d_8/OTP 95/$5_{w/w}$; (d) 16 mM TEKPOL in 3-PhPhOH. All spectra are recorded on a sample spinning at 10 or 12.5 kHz, at a temperature of ca. 100 K, and magnetic field of 9.4 T.

Supplementary tables

Table S1. Typical experimental parameters used to measure steady-state enhancement with ^{1}H-^{13}C CPMAS experiments.

^{1}H $\pi/2$ pulse	100 kHz (62.4W)
Number of pre-saturation pulses	15
Delay between pre-saturation pulses	3 ms
Recycle delay	20 s
CP spin-lock	^{1}H: ramp from 75 kHz to 80 kHz<br>^{13}C: 56 kHz
CP contact time	2 ms
μwave source	Gyrotron
Number of scans	μwave ON: 64<br>μwave OFF: 2048
Acquisition points	1014
Dwell time	9.8 μs
^{1}H decoupling	SPINAL-64 at 100 kHz
MAS rate	12.5 kHz

Table S2. Typical experimental parameters used to measure steady-state enhancement with ^{1}H-^{15}N CPMAS experiments.

^{1}H $\pi/2$ pulse	100 kHz (62.4W)
Number of pre-saturation pulses	15
Delay between pre-saturation pulses	3 ms
Recycle delay	20 s
CP spin-lock	^{1}H: ramp from 51 kHz to 57 kHz<br>^{15}N: 130 W
CP contact time	500 μs
μwave source	Gyrotron
Number of scans	μwave ON: 16<br>μwave OFF: 256
Acquisition points	2048
Dwell time	12.3 μs
^{1}H decoupling	SPINAL-64 at 100 kHz
MAS rate	12.5 kHz
